# Two fully automated data-driven 3D whole-breast segmentation strategies in MRI for MR-based breast density using image registration and U-Net with a focus on reproducibility

**DOI:** 10.1186/s42492-022-00121-4

**Published:** 2022-10-11

**Authors:** Jia Ying, Renee Cattell, Tianyun Zhao, Lan Lei, Zhao Jiang, Shahid M. Hussain, Yi Gao, H.-H. Sherry Chow, Alison T. Stopeck, Patricia A. Thompson, Chuan Huang

**Affiliations:** 1 Department of Biomedical Engineering, Stony Brook University, Stony Brook, NY 11794 USA; 2 Department of Radiation Oncology, Renaissance School of Medicine, Stony Brook University, Stony Brook, NY 11794 USA; 3 Department of Medicine, Northside Hospital Gwinnett, Lawrenceville, GA 30046 USA; 4 Program of Public Health, Renaissance School of Medicine, Stony Brook University, Stony Brook, NY 11794 USA; 5 Department of Radiology, Renaissance School of Medicine, Stony Brook University, Stony Brook, NY 11794 USA; 6 Department of Biomedical Informatics, Renaissance School of Medicine, Stony Brook University, Stony Brook, NY 11794 USA; 7 School of Biomedical Engineering, Health Science Center, Shenzhen University, Shenzhen, 518060 China; 8 University of Arizona Cancer Center, Tucson, AZ 85719 USA; 9 Department of Medicine, Renaissance School of Medicine, Stony Brook University, Stony Brook, NY 11794 USA; 10 Stony Brook Cancer Center, Stony Brook University, Stony Brook, NY 11794 USA; 11 Department of Medicine, Cedar Sinai Cancer, Cedars Sinai Medical Center, Los Angeles, CA 90048 USA

**Keywords:** Breast cancer, Breast density, Breast segmentation, Image registration, Deep learning

## Abstract

Presence of higher breast density (BD) and persistence over time are risk factors for breast cancer. A quantitatively accurate and highly reproducible BD measure that relies on precise and reproducible whole-breast segmentation is desirable. In this study, we aimed to develop a highly reproducible and accurate whole-breast segmentation algorithm for the generation of reproducible BD measures. Three datasets of volunteers from two clinical trials were included. Breast MR images were acquired on 3 T Siemens Biograph mMR, Prisma, and Skyra using 3D Cartesian six-echo GRE sequences with a fat-water separation technique. Two whole-breast segmentation strategies, utilizing image registration and 3D U-Net, were developed. Manual segmentation was performed. A task-based analysis was performed: a previously developed MR-based BD measure, MagDensity, was calculated and assessed using automated and manual segmentation. The mean squared error (MSE) and intraclass correlation coefficient (ICC) between MagDensity were evaluated using the manual segmentation as a reference. The test-retest reproducibility of MagDensity derived from different breast segmentation methods was assessed using the difference between the test and retest measures (Δ_2-1_), MSE, and ICC. The results showed that MagDensity derived by the registration and deep learning segmentation methods exhibited high concordance with manual segmentation, with ICCs of 0.986 (95%CI: 0.974-0.993) and 0.983 (95%CI: 0.961-0.992), respectively. For test-retest analysis, MagDensity derived using the registration algorithm achieved the smallest MSE of 0.370 and highest ICC of 0.993 (95%CI: 0.982-0.997) when compared to other segmentation methods. In conclusion, the proposed registration and deep learning whole-breast segmentation methods are accurate and reliable for estimating BD. Both methods outperformed a previously developed algorithm and manual segmentation in the test-retest assessment, with the registration exhibiting superior performance for highly reproducible BD measurements.

## Introduction

Breast cancer is the most common cancer among women worldwide and the leading cause of female cancer-related death for over three decades [1, 2]. Recent data show that female breast cancer treatment accounts for the highest total cancer-related medical care costs in the United States ($26 billion) with a projected increase of 30%-40% by 2030 [3]. More precise estimates of individual cancer risk are critical not only for improving the use of early intervention strategies (i.e.*,* enhanced screening and use of endocrine therapies for prevention) but also for reducing the economic burden of breast cancer, especially costs associated with late-stage disease.

Breast density (BD) is a radiologic measure of the proportion of fibroglandular tissue in the breast. A study in 2017 of more than 202000 women demonstrated that the presence of higher BD is the most prevalent risk factor for breast cancer [4]. As a significant and validated risk factor for breast cancer, BD has now been incorporated into breast cancer risk models to assess individual patients’ risk of developing cancer [5, 6]. Additionally, BD change has been examined in clinical trials as a surrogate measure for evaluating the efficacy of endocrine therapies, such as tamoxifen and aromatase inhibitors, for the prevention and treatment of breast cancer [7–10]. In a previous study, a ≥ 10% decrease in mammographic BD (MG-BD) after 12-18 mo of tamoxifen therapy was associated with clinical benefit as a reduction in the risk of breast cancer [11]. This finding suggests that BD is a modifiable risk factor, and a sensitive measure can enable earlier assessment of interventions aimed at lowering cancer risk and can offer a strategy for monitoring individual responses to endocrine therapy.

Currently, mammography is the predominant clinical method for BD assessment. The reliability of BD measurements with this modality is, however, compromised by the inherent limitations of two-dimensional projection. Even when bolstered by digital detector/image enhancement algorithms or tomosynthesis, mammography often fails to reflect the exact status of dense breast tissue [12] and presents risks related to radiation exposure if used frequently. Furthermore, clinical determination of MG-BD using the Breast Imaging Reporting and Data System classifies BD into only four descriptive categories (i.e., almost entirely fatty, scattered areas of fibroglandular density, heterogeneously dense, and extremely dense), which are highly subjective [13] with significant variation in intra- and inter-rater reliability [14]. Moreover, it is important for BD measurement to be reproducible, especially for longitudinal assessment of small to moderate changes in BD over time in an individual patient [15]. A more reproducible and accurate BD measure can assist clinicians in relating BD to individual breast cancer risks in clinical practice. It can also allow the assessment of BD changes in response to interventions earlier, with a smaller sample size and shorter trial duration, which could accelerate the drug development process [15]. Thus, a quantitatively accurate and highly reproducible BD measurement sensitive to small changes is desired.

Breast magnetic resonance imaging (MRI) is an excellent modality for quantifying BD because of its unique advantages including three-dimensional (3D) capability that circumvents breast compression, strong visual contrast between fibroglandular and fatty tissues, and absence of exposure to ionizing radiation. Previous studies demonstrated the potential of MR-derived quantitative BD assessments [16–19]. In these studies, standard T1-weighted MRI was used for BD evaluation; however, none of the studies considered representing true breast tissue composition [20], and the reproducibility of the MR-derived BD measures has not been well studied. Conversely, fat-water decomposition MRI is particularly useful due to its ability to separate MRI signals from protons in water and fat [21–23]. Our group has previously proposed a quantitative BD measure pipeline [24] based on whole-breast segmentation and fat-water decomposition MRI. In this pipeline, breast region is first identified using a dynamic programming breast segmentation method, and the proportion of fibroglandular content in the breast region is quantified using the fat fraction map (representing the relative percentage amount of fat signal in each voxel) derived from the fat-water decomposition MRI to obtain MR-based BD-MagDensity. This technique has been successfully used in multiple R01 clinical trials (NCT01761877, NCT02028221, and NCT04542135) [15, 25]. However, after applying the pipeline to a wider population of patients, it became apparent that the previous dynamic programming segmentation method could not always reliably exclude all pectoral muscles; particularly when the chest wall sharply jutted into the breast region, which is often observed in older women. This inconsistency in turn affects the accuracy and reproducibility of BD measurements. In order for the MR-based BD to be clinically useful, both accuracy and reproducibility are essential.

The purpose of this study was to develop an accurate and reproducible whole-breast segmentation method for the generation of highly reproducible MR-based BD measurements. In this study, two fully automated whole-breast segmentation algorithms based on image registration and deep learning were proposed. MagDensity was used as the quantitative BD measure model for task-based analysis because it has been proven as a reliable BD measurement and is comparable with the current standard (i.e., MG-BD). The performance of the segmentations was evaluated by directly assessing the accuracy and reproducibility of MagDensity.

## Methods

### Datasets

This study was approved by the local institutional review board, and written informed consent was obtained from all volunteers. The data used in this study were obtained from participants enrolled in two cancer prevention clinical trials assessing sulindac (NCT01761877; images acquired on 3 T Siemens Biograph mMR and Prisma) and metformin (NCT02028221; images acquired on 3 T Siemens Skyra) for their effect on BD. Three subsets of data were collected (Table 1).

- Data in the dictionary set were used to build a template dictionary for the registration breast segmentation method. A total of 28 single-sided breasts from the Sulindac trial were included in the dictionary, which contained different breast shapes, sizes, and densities. All single-sided breast image volumes included in the dictionary had no major fat-water swap artifacts or implants.
- Data from the deep learning set were used to develop and test the deep learning algorithm. This set included 28 breast volumes from the dictionary set and an additional 344 single-sided breasts from the sulindac and metformin trials, resulting in a total of 372 single-sided breast volumes.
- Data in the test-retest set were used to evaluate the test-retest reproducibility of MagDensity derived using different segmentation methods. Sixteen subjects from the Sulindac trial were randomly enrolled based on their availability and willingness to undergo repeated scans. Three of them had two test-retest scan sessions; therefore, 19 test-retest scan pairs were included. The data in the test-retest set were independent of the other datasets. The sulindac trial included patients who had a history of breast cancer, with images available from at least one unaffected breast with no breast implants. For this dataset, only the unaffected side of the breast was studied.

**Table 1:** Summary characteristics of the data used in different subsets

	Dictionary set	Deep learning set	Test-retest set
Purpose	To develop the template dictionary for the registration breast segmentation	To develop the deep learning network for the breast segmentation	To evaluate the test-retest reproducibility of MagDensity derived using available segmentation methods
Scanner	B, P	B, P, S	B, P
Data set	Sulindac	Sulindac and metformin	Sulindac

*B* Biograph mMR, *P* Prisma, *S* Skyra

### Breast MRI acquisition

3D Cartesian six-echo gradient echo MRI sequences were performed using dedicated Sentinelle 8-channel breast coils with the following parameters: repetition time = 30 ms; echo times = 1.37, 2.66, 4.92, 6.15, 7.38, and 8.81 ms, flip angle = 6°; matrix = 192 × 78; pixel size = 1.97 × 1.97 mm^2^, and slice thickness = 4 mm. Magnitude and phase images were collected. Test-retest scans were performed by the same technician on the same day for each participant in this group. After the first scan, the patient was asked to exit the scanner and immediately return to the scanner to receive the second scan. This ensures that changes related to plasticity or development are negligible, and therefore, intra-individual variation between the two scans can be considered as noise [26].

### Fat-water separation

The magnitude and phase images extracted from the scanner were combined to generate complex images using MATLAB (complex image = magnitude image × exp.(i × phase image)). Given that bipolar readouts were used in the acquisition, phase correction was performed, as previously described [27]. To reconstruct fat-only images, water-only images, and fat fraction maps, a multi-point Dixon method was applied by using a technique termed as “iterative decomposition of water and fat with echo asymmetric and least-squares estimation”, which incorporates asymmetrically acquired multi-echo data with an iterative least-squares decomposition algorithm with optimized noise performance [27, 28]. The fat-water separation process was performed using MATLAB R2020b (MathWorks, Natick, MA, USA).

### Algorithm 1: registration breast segmentation

#### Image processing

To suppress the background noise present in the breast MR images, the fat-only and water-only images were first combined to generate fat-water-sum image by adding their signal intensity values. In the fat-water-sum image, structural variations within the breast and pectoral regions were largely removed, making the breast region more homogeneous, which facilitated the generation of a mask containing the whole-body region only for the background noise removal process. To improve the visibility of the pectoral muscle and breast boundary, the fat-only and water-only images were combined using the sigmoid decision function [29]:

1 $${SI}_C\left(x,y,z\right)=\frac{1}{1+\exp \left(\frac{-{SI}_W\left(x,y,z\right)-0.25}{SI_F\left(x,y,z\right)}\right)}$$

where *SI*_C_*(x, y, z)*, *SI*_*W*_*(x, y, z)*, and *SI*_*F*_*(x, y, z)* denote the signal intensity values at positions *x*, *y*, and *z* of the combined, water-only, and fat-only images, respectively. In this fat-water-combined image, as demonstrated in the study by Wengert et al. [29], improved contrast and shape details of the tissues were evident, particularly in the area where the breast and pectoral muscle interface and comprise the posterior borders of the breast.

**Fig. 1:**
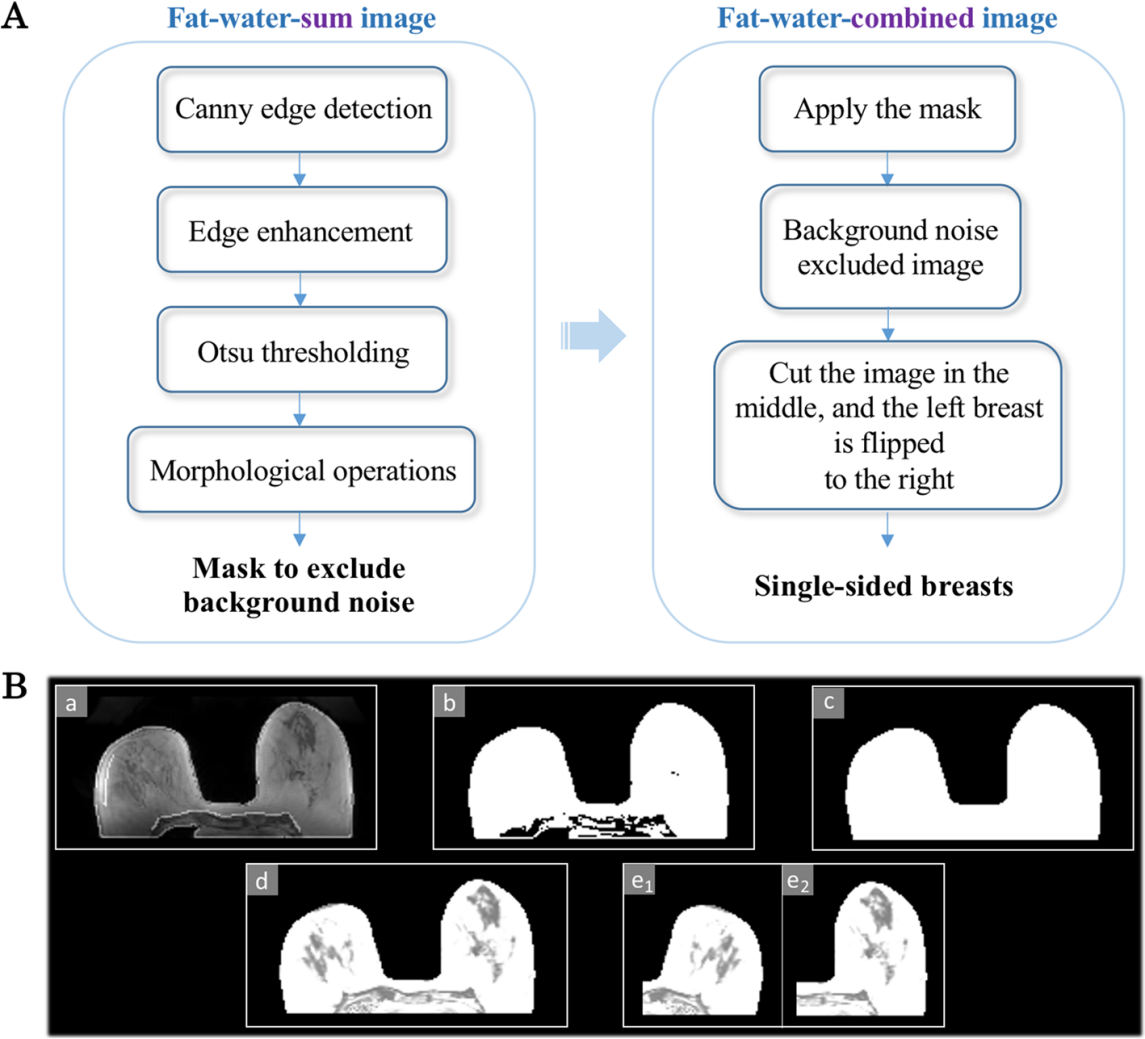
**A**. Workflow of the image processing steps for the registration breast segmentation method; **B**. The step-by-step results of the image processing. (a) Edge-enhanced fat-water-sum image after Canny edge detection method, (b) outcome of the Otsu thresholding, (c) mask after morphological operations, (d) body region extracted by applying the mask, and (e_1_) and (e_2_) single-sided breasts generated from D by cutting the breasts in the middle and flipping the left side to the right

The image processing pipeline is shown in Fig. 1A, and the corresponding results are shown in Fig. 1B. The fat-water-sum image data were normalized to a range from 0 to 1 using the min-max technique. Canny edge detection [30] was applied to the normalized fat-water-sum image. The intensity of the detected edge information was halved and added to the normalized fat-water-sum image to create an edge-enhanced fat-water-sum image. The Otsu’s thresholding technique [31] was applied to separate the image pixels into foreground and background (noise) classes. To generate a mask for the body region, morphological closing (dilatation followed by erosion) and filling operations were performed to fill the gaps [32]. By applying the mask to the fat-water-combined image, background noise was removed. Each generated image was divided into two individual images along the central vertical line, and the left breast image was flipped horizontally. Identically orienting breast images served the purpose of reducing the complexity of image registration to improve reproducibility. All procedures in this section were conducted using in-house software in MATLAB R2020b (MathWorks, Natick, MA, USA).

#### Dictionary building for registration approach

A template dictionary was built to serve as the basis for the registration process. As previously mentioned, after image processing, 28 single-sided breast images of various sizes and shapes were obtained. Their associated segmentations were manually delineated by precisely tracing the breast boundaries on the water images with the additional aid of the fat image, if needed, according to the manual tracing protocol reported by Rosado-Toro et al. [33] using ITK-SNAP (version 3.8.0). Specifically, in the transverse view, the two points where the anterior border starts to slope are considered as the starting points for the anterior border of the breast, and the posterior border is the interface between the pectoral muscle and breast tissue traced from these starting points. Manual tracing was conducted by a PhD student with 2 years of experience in breast image analysis under the guidance of a radiologist with 10 years of experience in breast imaging. A template image with its manual mask was considered as a template pair. A total of 28 template pairs were included in the dictionary.

#### Registration and segmentation

**Fig. 2:**
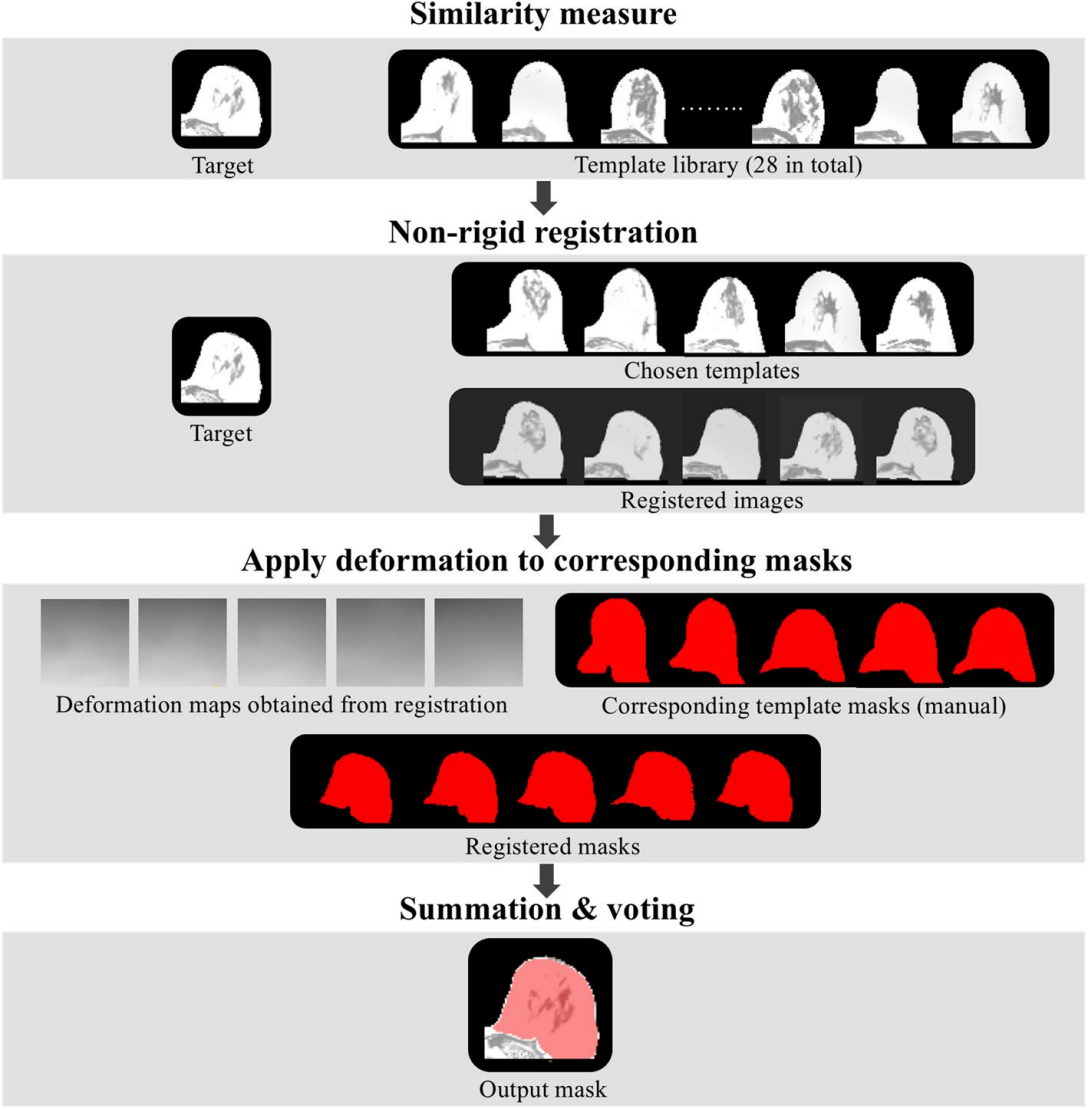
Pipeline of the registration breast segmentation algorithm. (1) Similarity measurement to choose the most similar five templates; (2) Registration of chosen template images to the target image and generation of deformation maps; (3) Application of the deformation maps to the corresponding template masks; (4) Summation of all the registered masks and voting to output the final segmentation of the breast (voxels contained in at least four registered template masks were included)

Figure 2 shows the pipeline of the registration breast segmentation algorithm. For each new dataset, the normalized mutual information (NMI) value was used as a similarity measure between the breast image volume of the new subject (target) and each template breast image volume in the dictionary. We empirically determined that the five template image volumes that achieved the highest NMI values were chosen for the next step. Non-rigid registration was performed from each selected template image volume to the target image volume. The deformation field of each selected template volume was then applied to its corresponding mask, resulting in five deformed masks. Only voxels contained in at least four deformed template masks were included in the final segmentation of the target breast. The entire procedure was implemented using MATLAB R2020b (MathWorks, Natick, MA, USA) and NiftyReg package [34]. The algorithm was applied to the test-retest dataset.

### Algorithm 2: deep learning breast segmentation

#### Image processing

Deep learning algorithm is a powerful alternative for whole-breast segmentation. Data in the deep learning subset (372 single-sided breast volumes) were randomly split into training, validation, and testing sets (222, 74, and 76 volumes, respectively). After fat-water decomposition was performed, as mentioned previously, min-max normalization was applied to the image data. Breast segmentations of these image volumes were generated using previously published automated dynamic programming segmentation [24]. For those with issues at the posterior border of the breast region, manual correction was performed using ITK-SNAP (version 3.8.0). Similar to the registration algorithm, the fat-only and water-only images were divided in the middle, and the left side was flipped to the right.

#### 3D U-Net architecture

**Fig. 3:**
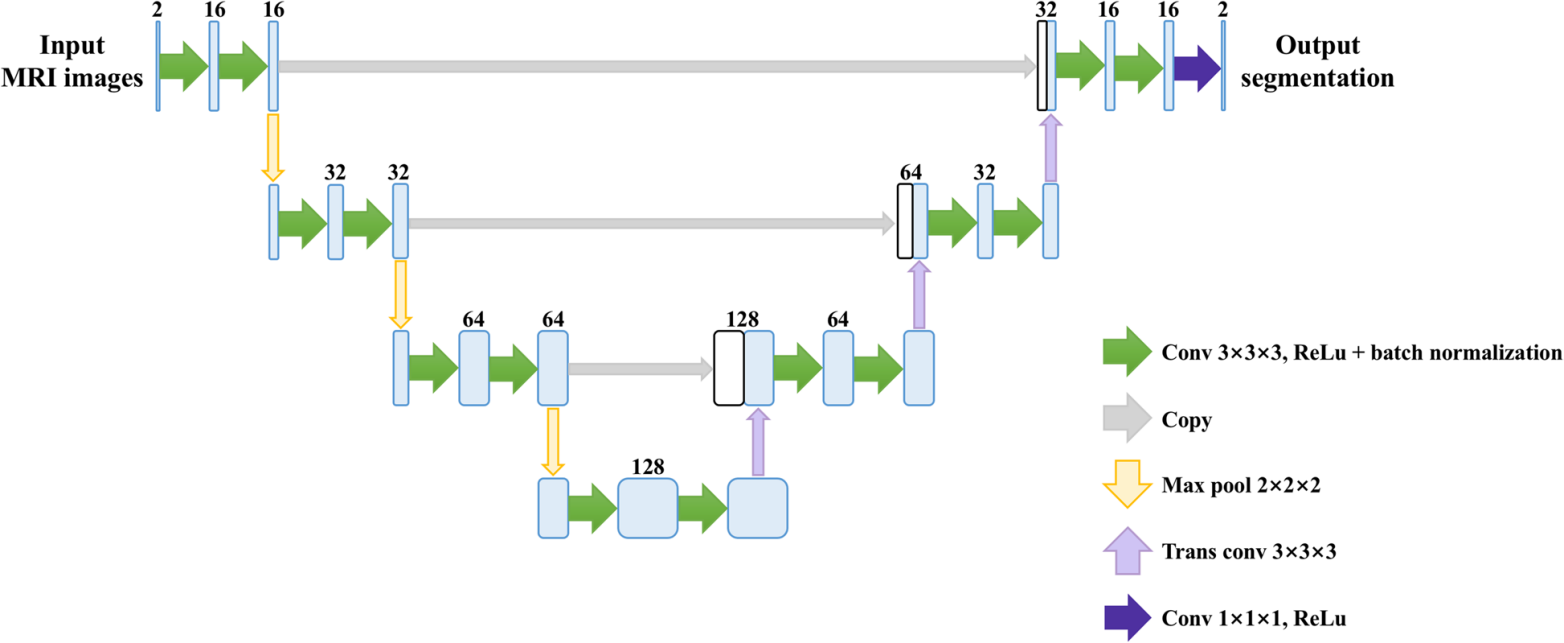
Architecture of 3D U-Net for deep learning breast segmentation. The input contains two channels, one for water-only image and the other for fat-only image. The output is segmentation map. The blue boxes represent feature maps. The white boxes are copied feature maps. The number of channels is labeled on the top of the box. Different arrows denote different operations that are indicated in the legends

A 3D U-Net was implemented based on the U-Net architecture [35] to automatically segment the breast region. In our work, different variants were used, and batch normalization was added to normalize the input to the next layer, making the training less sensitive to weight initialization and accelerating the training process [36]. The details of the architecture are shown in Fig. 3. The input of the network contained two channels: fat- and water-only images. The contracting path (left side) included repeated application of two 3 × 3 × 3 padded convolutions with a stride of 1; each followed by a rectified linear unit (ReLU) activation function and batch normalization. Each repeated operation was followed by a 2 × 2 × 2 max pooling layer with a stride of 1. The expansive path (right side) consisted of one 2 × 2 × 2 transposed convolution with a stride of 1, followed by concatenating the feature map of the same size from the contracting path, and two 3 × 3 × 3 padded convolutions with a stride of 1; each followed by a ReLU and batch normalization. The final layer was a 1 × 1 × 1 convolution with a ReLU. All operations were performed in 3D. One Titan Xp and one RTX 2070, with TensorFlow 1.14.3 and Ubuntu 18.04.4, were used together to develop the model. The batch size was set to three based on the limit of the GPU memory (Titan Xp: 12GB; RTX 2070: 8GB). The Adam optimizer was used as it is computationally efficient (less memory required) and exhibits good generalization performance [37]. If the validation loss did not improve within 50 epochs, the learning rate would decrease to 0.33 times the last learning rate. The learning rate stopped decreasing once it hit 10^−5^. Training was stopped if the validation loss did not improve within 200 epochs. These parameters were tuned based on the validation loss. The best model in terms of validation loss was saved. The tasks described in this section were implemented using Python.

#### Post processing

The network output pixel-wise probability maps indicate the likelihood of a pixel belonging to the breast. Thresholding was performed to remove low possibility results. The threshold value was selected based on the Dice coefficient of the training and validation data, and the final threshold was 0.35. Hole filling was performed using a MATLAB morphological filling operation [32]. The trained deep learning model was applied to the test-retest subjects.

### Previously published dynamic programming breast segmentation

The dynamic programming breast segmentation approach published previously [11] implemented an upgraded k-means++ classification method combined with a 3D regulation to achieve the goal of whole-breast segmentation. It was performed on the test-retest set, and the segmentation results were further analyzed for comparison purposes.

### Manual breast segmentation for validation

To assess the intra- and inter-rater reliabilities, two versions of manual segmentations were drawn on the test-retest dataset by a radiologist resident and a graduate student under the guidance of a radiologist with more than 10 years of experience in breast imaging based on the guidelines reported in the study by Rosado-Toro et al. [33] using ITK-SNAP (version 3.8.0).

### Task-based analysis and statistics

In this study, MagDensity was selected as the quantitative BD measure model to assess the segmentation performance. It was calculated using all four segmentation methods: registration, deep learning, dynamic programming, and manual. To evaluate the concordance of MagDensity derived from the latter three automated segmentations and the manual segmentation drawn by the radiologist, the mean squared error (MSE) and intraclass correlation coefficient (ICC) were calculated. The test-retest reproducibility of MagDensity derived from the breast segmentation methods was evaluated using the difference between the test and retest measures (Δ_2-1_), MSE, and ICC. Specifically, the two-way mixed, single-measure ICC model was chosen to assess the absolute agreement ICC. An ICC of ≥ 75% was considered excellent agreement, whereas 40%-74% as fair-to-good. Analyses were performed using MATLAB R2020b (MathWorks, Natick, MA, USA) and IBM SPSS Statistics (version 27).

## Results

### Comparison of breast segmentation results

**Fig. 4:**
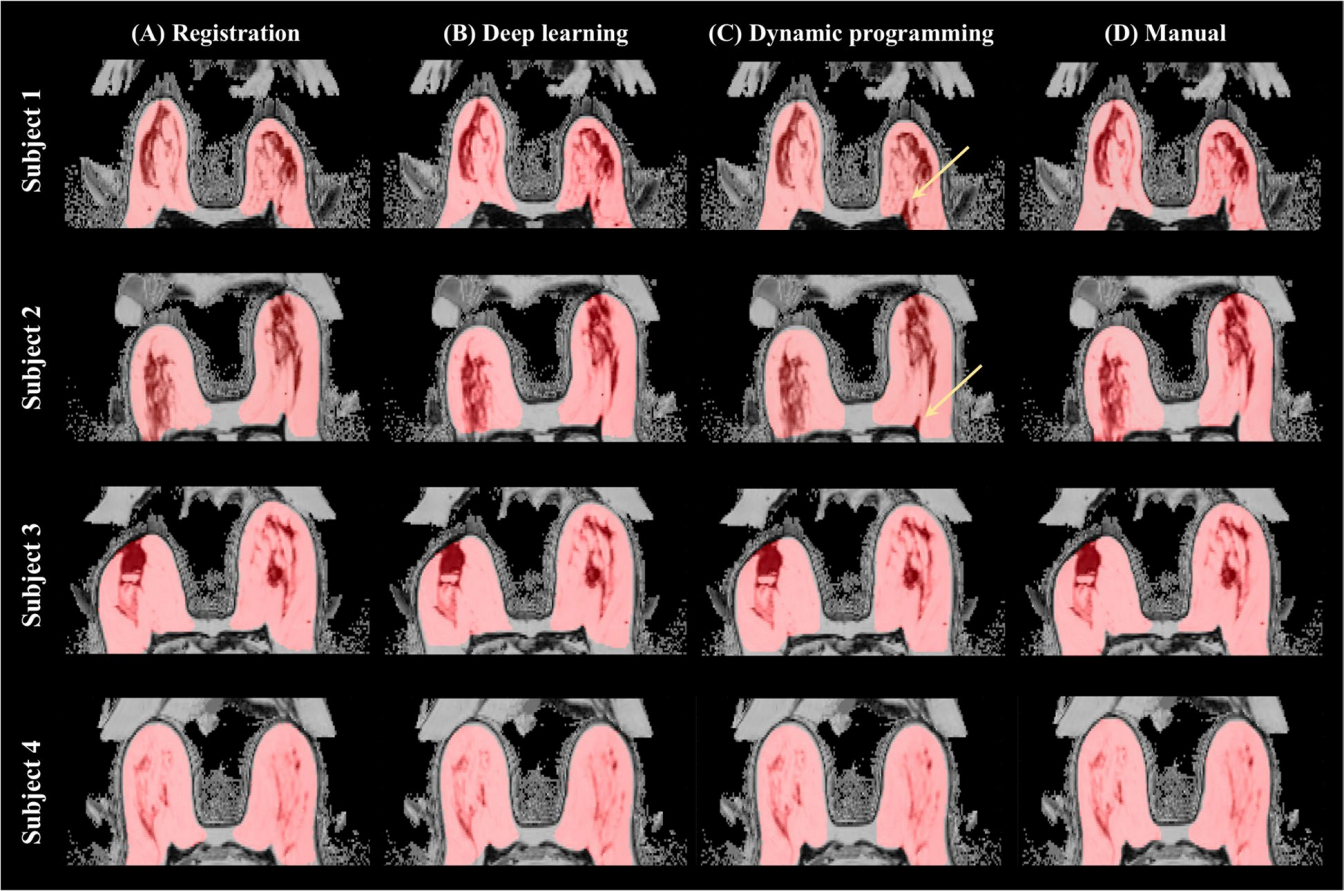
Representative examples of whole-breast segmentation results using **A** registration, **B** deep learning, **C** dynamic programming, and **D** manual methods

Figure 4 shows four representative examples of the segmentation results from registration, deep learning, dynamic programing, and manual methods. As shown, the registration algorithm exhibited higher accuracy in depicting the posterior breast boundaries.

### Concordance of MagDensity derived from automated and manual segmentations

In this analysis, the manual segmentation obtained from the radiologist was used as the reference standard. Table 2 shows the concordance of the MagDensity derived using automatically generated and manually delineated segmentations. MagDensity derived using the three automated breast segmentation models (registration, deep learning, and dynamic programming) exhibited strong absolute agreement with that derived from the manual segmentation (ICCs ≥0.975), while the registration model showed the smallest MSE (0.693, *small is better*) and highest ICC (0.986, *large is better*).

**Table 2:** Concordance of MagDensity derived using the automated algorithms and manual segmentation (reference standard)

	Registration	Deep learning	Dynamic programming
**MSE**	0.693	0.781	1.124
**ICC** **95%CI**	0.986 (0.974, 0.993)	0.983 (0.961, 0.992)	0.975 (0.869, 0.991)

### Test-retest reproducibility

**Fig. 5:**
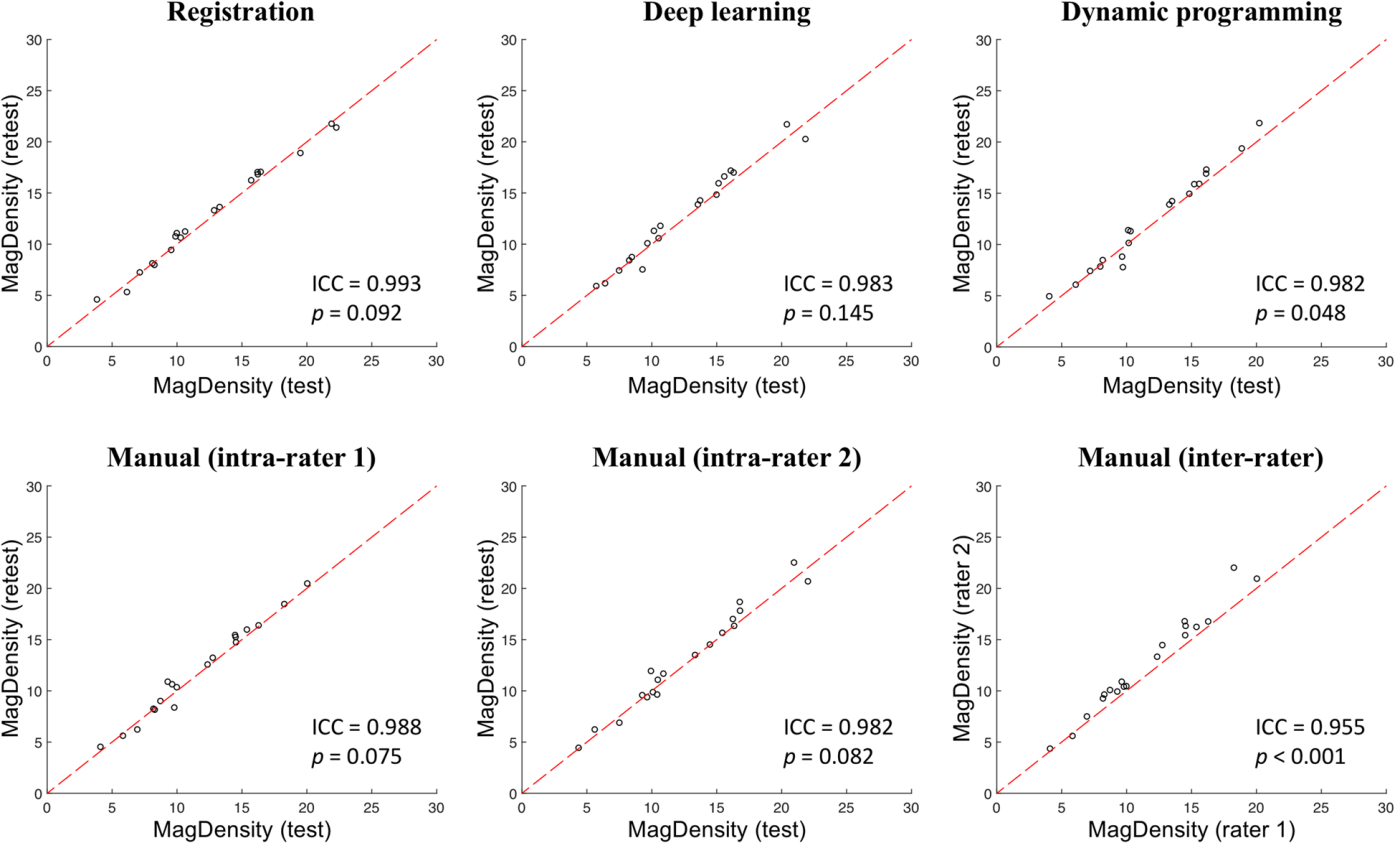
Plots of test-retest results of MagDensity derived using different segmentation methods. The red dashed line indicates the line of agreement. The quantitative assessment is shown in Table 3

**Fig. 6:**
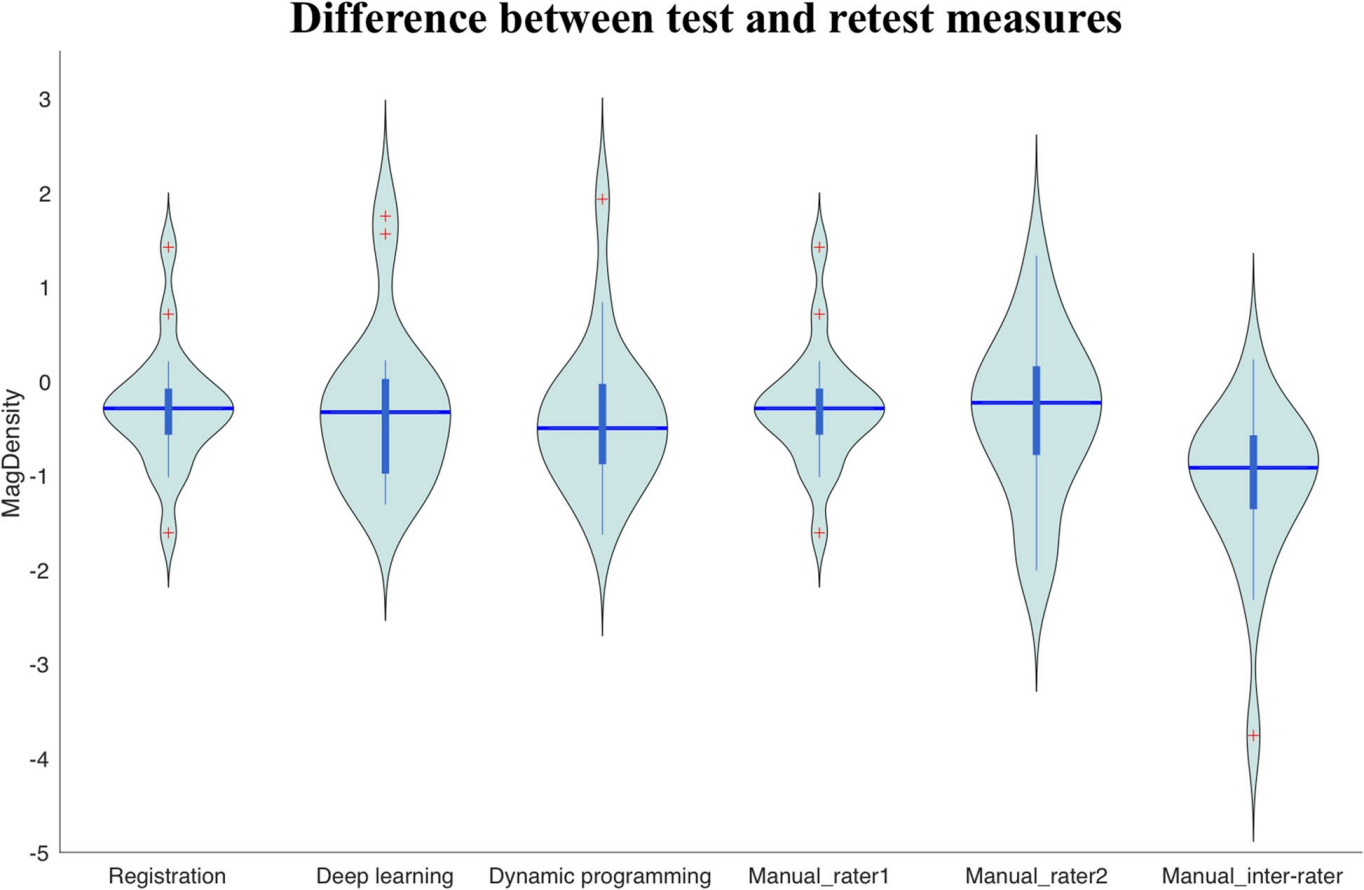
Violin Plots of test-retest measures of MagDensity derived using different segmentation methods. The quantitative assessment is shown in Table 3

The test-retest reproducibility of MagDensity derived using the four different segmentation methods is summarized in Table 3, and the corresponding plots are presented in Figs. 5 and 6. Both algorithms proposed in this study showed high test-retest reproducibility in the MagDensity measure with ICCs ≥ 0.983. Among the algorithms investigated, the results obtained with the registration algorithm showed the smallest difference between the test and retest measures and smallest MSE (0.370) and achieved the best ICC (0.993; 95%CI: 0.982-0.997).

**Table 3:** Comparison of test-retest reproducibility of MagDensity derived using different segmentation methods

	Registration	Deep learning	Dynamic programming	Manual intra-rater (rater 1)	Manual intra-rater (rater 2)	Manual inter-rater (rater 1 vs rater 2)
Mean Δ_2-1_	**0.236**	0.292	0.390	0.282	0.369	1.116
Mean |Δ_2-1_|	**0.529**	0.683	0.695	0.540	0.702	1.140
Max |Δ_2-1_|	**1.094**	1.748	1.938	1.615	2.014	3.763
MSE	**0.370**	0.741	0.763	0.479	0.855	1.967
ICC (95%CI)	**0.993** **(0.982, 0.997)**	0.983 (0.956, 0.993)	0.982 (0.949, 0.993)	0.988 (0.966, 0.995)	0.982 (0.952, 0.993)	0.955 (0.444, 0.989)

∆_2 − 1_: Difference between test-retest measures

## Discussion

In this study, we investigated two fully automated whole-breast segmentation approaches, based on image registration and deep learning, to better assist studies monitoring longitudinal changes in BD. MagDensity [24] was selected as the quantitative MR-based BD measure for the task-based analysis because it has been shown to be reliable and directly comparable with MG-BD when accurate breast segmentation is provided.

**Fig. 7:**
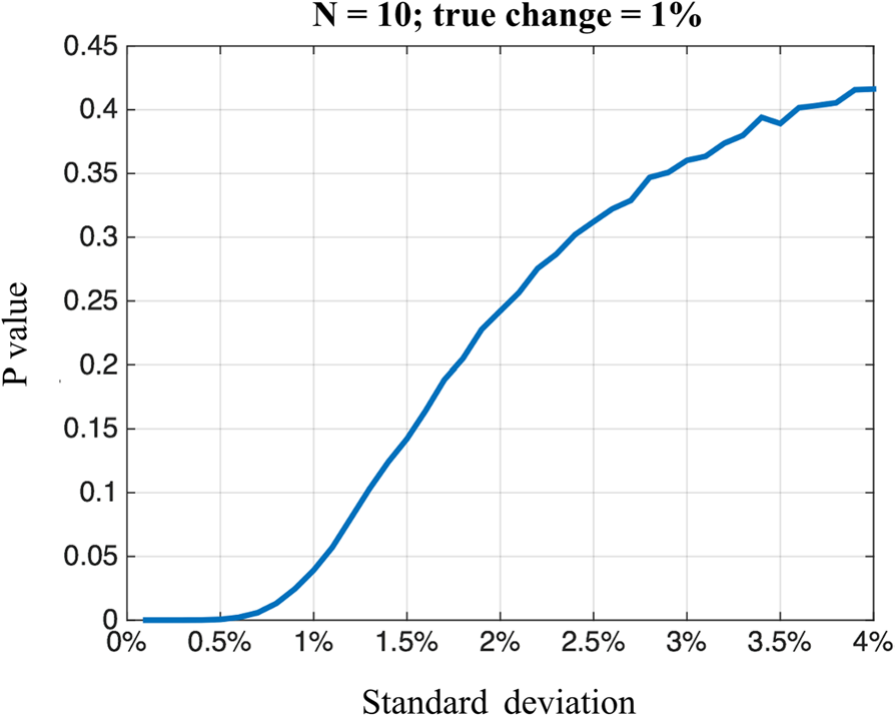
A simulation illustrating the statistical significance of a small enhancement of BD measurement reliability. When the test-retest standard deviation is reduced from 1.42% to 1.11%, the *p* value decreases from 0.13 to < 0.05 (assume *N* = 10; true change = 1%)

Our work demonstrates that both proposed segmentation algorithms are highly concordant with the manual segmentation obtained from the experienced radiologist in calculating the MR-based BD. Moreover, the registration breast segmentation method has yielded the smallest variability and highest ICC value in the test-retest analysis, indicating that this algorithm is highly reproducible for BD measurements. This is highly desirable as illustrated by a simulated experiment shown in Fig. 7, when the test-retest standard deviation is reduced from 1.42% to 1.11% (assuming *N* = 10 and true change = 1%), the *p* value decreases from 0.13 to < 0.05. In addition to removing the reliance on manual methods, this study demonstrates that small improvements in the reliability of breast segmentation can dramatically enhance the ability to detect small changes in BD, which further reduces the sample size, duration, and cost of clinical trials evaluating changes in BD [15].

In recent years, a number of breast MRI segmentation methods have been introduced [29, 38–42]. Lin et al. [41] proposed a two-step template-based model to achieve 3D breast segmentation. The authors manually identified three body landmarks on a middle slice to create a subject-specific chest template to exclude the chest region and define the posterior border for breast segmentation, this reference template was later applied to determine breast boundaries in adjacent slices to generate 3D results [41]. The idea of the subject-specific template is attractive; however, one of the cited landmarks is located at the thoracic spine, which is commonly excluded from the field of view of most breast MRIs, thus significantly limiting its application. A similar limitation was observed in the localized-atlas-based segmentation model reported by Fooladivanda et al. [38]. To achieve breast segmentation for BD estimation, Wengert et al. [29] generated nine template models as the reference wherein the algorithm automatically selected the most similar model to perform image registration until it matched the targeted breast. However, the study did not clarify the measure used to determine the similarity, and the sample size in the reference pool seemed to be very limited given that individuals’ breasts can vary significantly in terms of shape, size, and the distribution of fibroglandular tissues.

Another common issue in breast segmentation is that it is challenging for an automated segmentation algorithm to precisely differentiate the interface of the breast tissue and pectoral muscle due to its widely varying appearance across individuals [38, 43]. The previously published dynamic programming model encountered similar difficulties in identifying posterior boundaries, especially regarding images that featured pectoral muscle borders sharply ‘jutting’ into the breast region (Fig. 4C). Our proposed algorithms (registration and deep learning) successfully solved this issue and presented accurate posterior borders as indicated in Fig. 4A and B.

Furthermore, the approaches presented in this study can be easily extended. For the registration model, if a ‘problematic’ segmentation occurs due to lack of similar cases in the template dictionary, the segmentation result may be manually corrected and added to the dictionary. Similarly, the training set in the deep learning algorithm can be extended to facilitate model learning and improve the accuracy of future segmentation. Moreover, two versions of manual segmentation were obtained for comparison purposes. Manually delineating breast regions for a large amount of data is time-consuming; drawing masks for a bilateral breast volumes takes approximately 30 to 45 mins. This limitation not only results in intra- and inter-rater variability but also restricts the possibility of large-scale application, and thus, it is clinically impractical. Conversely, the registration and deep learning methods can generate efficient measurements within seconds, which is favorable for practical applications.

When comparing the two proposed approaches, the registration method exhibited superior accuracy and reliability in deriving MagDensity even with a much smaller dictionary (training set) size, whereas the deep learning algorithm required a larger amount of data to train the model to a reliable standard of performance. However, the results reported here only reflect the performance of both algorithms using our available training data. Although the deep learning algorithm did not perform as well as the registration algorithm in this study, it still exhibits potential for improvement if additional data are provided. Additionally, the underlying logic of the deep learning method involves learning from the training data and developing a model that can be used to accomplish the task, whereas the registration method relies on the non-rigid registration of the selected template images. This suggests that the registration algorithm is dependent on the accuracy of the registration process, and we assume that an algorithm (i.e., deep learning) understanding more of the underlying rationale would exhibit better performance for certain challenging cases (e.g., very dense breasts; no clear boundary between the fibroglandular tissue and pectoral muscle) that are not included in the test-retest dataset. Therefore, both algorithms are potentially useful for the purpose of breast segmentation.

A limitation of the current study is the exclusion of patients with breast implants. Taking into consideration that the two proposed breast segmentation methods may not be applicable in subjects presenting surgical implants, future studies should aim to explore segmentation methods that can accurately detect and exclude breast implants prior to the measurement of BD. Additionally, since test-retest data are extremely valuable, the data used for reproducibility assessment were randomly acquired from patients who are available and willing to participate in the research project; thus, the sample size is relatively limited. Moreover, given that both proposed breast segmentation algorithms were only conducted on images acquired using 3 T Siemens scanners (Biograph mMR, Prisma, and Skyra), further studies are needed to validate the strategies using a larger sample size across different vendors, scanners, centers, and field strengths (e.g., 1.5 T).

## Conclusions

In conclusion, the proposed registration and deep learning whole-breast segmentation strategies generate accurate and reliable breast segmentations that enable highly reproducible MR-based BD measurements, with the former segmentation method exhibiting superior performance even with a smaller training dataset size. The proposed breast segmentation methods, combined with MagDensity, present a valuable strategy for the precise evaluation of subtle longitudinal BD changes as an aid in the assessment of breast cancer prevention strategies, especially in postmenopausal women where changes are expected to be smaller in response to endocrine therapies.

## Data Availability

The data that support the findings of this study are available from the corresponding author, Chuan Huang, upon reasonable request.

## Abbreviations

BD: Breast density
MG-BD: Mammographic breast density
MRI: Magnetic resonance imaging
3D: Three-dimensional
NMI: Normalized mutual information
ReLU: Rectified linear unit
MSE: Mean squared error
ICC: Intraclass correlation coefficient
